# Hemodynamic Monitoring

**DOI:** 10.1155/2012/516979

**Published:** 2012-08-22

**Authors:** Antoine Vieillard-Baron, Anthony McLean, Paul Mayo, Philippe Vignon

**Affiliations:** ^1^Intensive Care Unit, University Hospital Ambroise Paré, Boulogne 92104, France; ^2^Department of Intensive Care Medicine, Western Clinical School, Nepean Hospital, Penrith, NSW 2751, Australia; ^3^Division of Pulmonary, Critical Care and Sleep Medicine, Long Island Jewish Medical Center, 410 Lakeville Road, New Hyde Park, NY 11040, USA; ^4^Medical-Surgical Intensive Care Unit, Dupuytren Teaching Hospital, Limoges 87042, France

This special issue is dedicated to hemodynamic monitoring. Papers include didactic papers and original clinical studies which originate from 6 countries in 3 continents (USA, Sweden, England, France, Portugal, and Australia). This reflects the worldwide interest of ICU physicians for this type of monitoring. This renewed interest for the hemodynamic monitoring is presumably due to the emergence of new technologies and concepts which can easily be implemented on clinical grounds by the intensivist to better guide acute therapy in shocked patients. New concepts, because functional hemodynamic monitoring and preload responsiveness, rather than the traditional preload assessment, are widely proposed as an attractive alternative to the conventional static approach of hemodynamic assessment. New technologies, because minimally or noninvasive techniques, such as, echocardiography, have yet gained wide acceptance and provide unparalleled information when compared to conventional invasive monitoring techniques (right-heart catheterization). For instance, critical care echocardiography has been defined as an echocardiography performed by intensivists themselves at the bedside to evaluate hemodynamics [1, 2]. 

This special issue emphasizes the recent conceptual evolution of hemodynamic monitoring in the ICU setting. Historically, hemodynamic monitoring was based on a quantitative approach which relied on static parameters obtained by invasive methods, whereas the modern approach is rather functional and based on dynamic indices derived from heart-lung interactions, especially in mechanically ventilated patients. Accordingly, this special issue may help the reader to understand step by step this in-depth conceptual evolution.

A. Napoli reviewed most of these concepts. In their contribution, S. Huang and A. Mclean focused on a practical approach of hemodynamic monitoring based on transthoracic echocardiography, while M. Chew proposed a larger routine use of echocardiography. P. Squara and C. Waldmann suggested that the development of new monitors allowing to less invasively measure cardiac output should be integrated in clinical practice to obtain an exhaustive, real-time, hemodynamic assessment which they coined “intelligent hemodynamic monitoring”. How to guide fluid resuscitation is also debated, either when using ScVO_2_ as suggested by S. Nebout and R. Pirracchio, or by testing the circulatory system to identify fluid responsiveness. L. Levitov and P. Marik reviewed the echocardiographic assessment of fluid responsiveness, using either the transthoracic or the transesophageal route. S. Préau et al. proposed to use pulse pressure variations and respiratory changes of peak velocity of Doppler arterial flow to identify potential fluid responders in spontaneously breathing patients during a standardized deep inspiration effort. F. Pissarra et al. reviewed the clinical value of transesophageal echocardiography in the operating room to optimize hemodynamics. R. Maharaj reviewed the clinical interest of extravascular lung water assessment in patients with acute lung injury.

Overall, the current special issue on hemodynamic monitoring emphasizes its clinical utility and describes currently available tools, while discussing the evolving concepts which mainly rely on the use of combined parameters to predict fluid responsiveness and on the assessment of both the efficacy and tolerance of blood volume expansion to guide fluid therapy and ultimately patient's acute care.

*Antoine Vieillard-Baron* *Anthony McLean* *Paul Mayo* *Philippe Vignon*
